# The interaction between RE1-silencing transcription factor (REST) and heat shock protein 90 as new therapeutic target against Huntington’s disease

**DOI:** 10.1371/journal.pone.0220393

**Published:** 2019-07-30

**Authors:** Raúl Orozco-Díaz, Angélica Sánchez-Álvarez, José Manuel Hernández-Hernández, José Tapia-Ramírez

**Affiliations:** 1 Department of Genetics and Molecular Biology, Centro de Investigación y de Estudios Avanzados-IPN, México City, Mexico; 2 Department of Cell Biology, Centro de Investigación y de Estudios Avanzados-IPN, México City, Mexico; Hokkaido Daigaku, JAPAN

## Abstract

The wild type huntingtin protein (Htt), supports the production of brain-derived neurotrophic factor (BDNF), a survival factor for striatal neurons, through cytoplasmic sequestering of RE-1silencing transcription factor (REST). In Huntington´s Disease an inherited degenerative disease, caused by a CAG expansion in the 5´coding region of the gene, the mutant huntingtin protein (mHtt), causes that REST enters pathologically into the nucleus of cells, resulting in the repression of neuronal genes including BDNF, resulting in the progressive neuronal death. It has been reported that Htt associates with Hsp90 and this interaction is involved in regulation of huntingtin aggregation. Discovering mechanisms to reduce the cellular levels of mutant huntingtin and REST provide promising strategies for treating Huntington disease. Here, we use the yeast two-hybrid system to show that N-terminus or REST interacts with the heat shock protein 90 (Hsp90) and identifies REST as an Hsp90 Client Protein. To assess the effects of Hsp90 we used antisense oligonucleotide, and evaluated the levels mHtt and REST levels. Our results show that direct knockdown of endogenous Hsp90 significantly reduces the levels of REST and mutant Huntingtin, decreased the percentage of cells with mHtt in nucleus and rescued cells from mHtt-induced cellular cytotoxicity. Additionally Hsp90–specific inhibitors geldanamicyn and PUH71 dramatically reduced mHtt and REST levels, thereby providing neuroprotective activity. Our data show that Hsp90 is necessary to maintain the levels of REST and mHtt, which suggests that the interactions between Hsp90-REST and Hsp90-Huntingtin could be potential therapeutic targets in Huntington's disease.

## Introduction

Huntington’s disease (HD) is an autosomal dominant neurodegenerative disorder caused by the mutant huntingtin protein (mHtt). The molecular basis of the disease lies in the expansion of CAG repeats in the first exon of the huntingtin gene (Htt), the mutant protein contains an abnormally long polyglutamine stretch at the N terminus [1]. Patients with HD present severe atrophy of the neostriatum wherein the most striking feature is neuronal loss in the striatum (caudate putamen) along with enlarged lateral ventricles [2]. Normal Htt (Htt) supports the production of a corresponding cytoplasmic protein, whereas N-terminal fragments of mHtt are accumulated in nuclei and induce neurodegeneration in patients with HD [3, 4]. Htt sustains the production of brain-derived neurotrophic factor (BDNF), a survival factor for striatal neurons, through cytoplasmic sequestering of repressor element-1 transcription factor/neuron-restrictive silencer factor (REST/NRSF) [5, 6], a major silencing transcription factor that regulates the expression of numerous neuronal genes, through its binding to a sequence of 21 base pairs, called repressor element 1 (RE1) [7].

Initially, REST target genes were thought to contain one to five RE1-binding elements within their promotor region. Later, the use of chromatin immunoprecipitation sequencing (ChIP-seq) revealed numerous other REST binding motifs besides the RE1-sequence, which increased the REST-dependent gene pool markedly [8–10]. Furthermore, presence of RE1-binding sites in potential target genes does not necessarily implicate REST-mediated repression, as only RE1-containing genes with midrange binding properties are subjected to selective REST regulation. Our brain requires a fine regulation, since it is the ultimate cellular and molecular regulator, it responds to conditions of stress, behavior and adaptations, whose final result may be resilience or vulnerability. In addition, stress-induced epigenetic chromatin remodeling is a major regulator of neuronal gene expression and influences phenotypic responses, i.e. resilience or vulnerability [11–13]

It is therefore not surprising that neurons have different mechanisms of security and defense to preserve neuronal function and intrinsic homeostasis against stressful insults, including injury, disease, and oxidative stress. In this sense REST ensures neuronal specificity and cell type-specific gene expression during brain development, and because REST´s epigenetic influence on genes critically involved in neuronal function, including genes that regulate synaptic plasticity, axonal growth, vesicular transport and ion conductance, REST is, therefore, an fine-tune neuronal gene that shapes neuronal homeostasis following stressful experiences.

Whilst REST is normally quiescent in differentiated neurons, the transcriptional repressor can be upregulated following various intrinsic and extrinsic neuronal insults. Following ischaemia, hypoxia and epileptic seizures REST nuclear upregulation generally appears to elicit a harmful response in the hippocampal neuronal through repression of synaptic signaling genes [14–17]. However, when neurodegenerative diseases occur, the mechanism looks different; REST levels decrease. In Alzheimer's disease REST decreases in Neurons [18, 19]. Similarly in Parkinson's disease, in dopaminergic neurons, REST decreases [20].

In Huntington´s disease, Htt indirectly regulates REST nuclear trafficking via the interaction of REST/NRSF-interacting LIM domain protein (RILP) and dynactin p150Glued*, an Htt-associated protein 1 (HAP1) bound to the Htt protein. The formation of this complex causes REST retention in the cytoplasm, in opposite way the binding of mHtt to this complex alters its conformation, permitting REST/NRSF to be inappropriately transported to nuclei [21]. A direct effect of this erroneous transport of REST is the repression of neuronal genes containing RE1 sequences, including the brain-derived neurotrophic factor (BDNF) [5, 6]. In Huntington´s disease models, mHtt further triggers a pathogenic cascade involving Sp1 activation, which leads to the upregulation of REST [22]. The weak interaction of mHtt with HIP1 protein also results in increased nuclear accumulation of HIPPI and HIP1, leading to occupancy of HIPPI at the REST promoter, triggering its transcriptional activation and directly repress REST target genes [23]. Hence, these genes are intimately involved in the regulation of many cardinal cellular processes for neuronal identity and function [24], and REST contributes to HD pathogenesis. Modulating the levels, transport o rate of synthesis and degradation of REST could be important for the possible treatment of Huntington´s disease. Interestingly, several research groups have focused their efforts on finding small molecules, which are capable of antagonizing REST/NRSF silencing activity, and other groups propose the use of alternative REST splicing to modulate REST levels [25].

Given the importance of REST and the complexity of its functions, we investigated its interacting partners that may influence its folding, stability, and function. Using the yeast Two-hybrid screening, we identified Hsp90 as an unprecedented REST interacting protein. Co-immunoprecipitation, His-tag pull-down assays were performed to demonstrate the REST-Hsp90 interaction. Additionally, in order to analyze the effect of Hsp90 inhibition on REST levels, assays with antisense oligos (ASO) and specific inhibitors of Hsp90 activity were designed and carried out. Our results show that knockdown or inhibition of endogenous Hsp90 by antisense oligonucleotides, GA or PU-H71 down-regulated REST/NRSF. Hsp90 is a heat shock protein that performs functions that control the activity, turnover, and trafficking of a variety of client proteins. Its inhibition alters the function and/or turnover of Hsp90-bound proteins, providing a rapid means for screening Hsp90-regulated targets [26]. Hsp90 is classified into Hsp90α and Hsp90β; the first is induced by stress, while the second is constitutively expressed, and both have similar functions [27, 28].

The interaction of Hsp90 with Htt has been previously described [29], and since Htt is a client protein of Hsp90, its pharmacological inhibition disrupts the corresponding interactions, induces clearance of mHtt through the ubiquitin-proteasome system [16], and thereby suppresses the formation of insoluble mHtt exon 1 aggregates [30].

In the present study, we found that mHtt acts within nuclei to induce cellular cytotoxicity by inducing apoptosis in differentiated SH-SY5Y cells. We also found that REST is a novel Hsp90 associated protein and that Hsp90 has a binding preference for mHtt versus Htt. Hsp90-specific inhibitors geldanamycin (GA) and PU-H71 inhibited the stability of mHtt and REST, providing excellent neuroprotective activity. The inhibition of Hsp90 expression by Hsp90 ASO decreased mHtt and REST levels, decreased the number of cells with mHtt in nucleus and the programmed cell death. So Hsp90 ASO had a protective effect against the cytotoxicity of mHtt.

Our data show that Hsp90 is necessary to maintain the levels of REST and mHtt, which suggests that the interactions between Hsp90-REST and Hsp90-Huntingtin could be potential therapeutic targets to Huntington´s disease.

## Material and methods

### Cell culture and differentiation

SH-SY5Y cells were cultured in Dulbecco’s modified eagle medium (DMEM) supplemented with 10% heat-inactivated fetal bovine serum (FBS), non-essential amino acids (0.1 mM), sodium bicarbonate (3.6 g/L), sodium pyruvate (1 mM), L-glutamine (2 mM), and antibiotics (100 U/ml penicillin and 100 U/ml streptomycin) in a 5% CO_2_ humidified incubator at 37°C. After 24 h of cell plating, differentiation was induced by lowering the FBS in culture medium to 3% and adding 10 μM all-trans retinoic acid (RA) (Sigma, R2625) and 0.5% B-27 supplement (Thermo Fisher Scientific, 17504044) during 4 days, changing the medium every day. To evaluate the differences in cell morphology in proliferative and differentiated cells, we analyzed cells under phase-contrast light microscopy using a 20x oil immersion objective. Images were captured with epifluorescence microscopy (Leica Microsystems, DMI6000CS). To validate our neuronal cell model, we used three neuron-specific markers; NeuN, beta III tubulin and NSE. We observe cells that acquire the neuronal phenotype in the presence of retinoic acid and express neuronal markers.

### Yeast two-hybrid screening

Plasmids containing amino terminal repressor domains (TRD1) were used as bait and 5 μg of HeLa cells cDNA library containing cDNA fragments inserted into the Eco RI and Xho I sites of the pGAD GH plasmid (pGAD GH-cDNA) was used as the source of prey proteins. Plasmid pGAD GH contains the *LEU2* gene, GAL4 Activation Domain (GAL4 AD), 2 μm ori, Amp^r^, and ColE1 ori. Library screens were carried out as previously described [31, 32] using HIS3 and lac Z reporters. The S*accharomyces cerevisiae* L40 strain (MATa: Trp^-^, Leu^-^, His^-^, LYS2::lexA-HIS3, URA::lexA-lacZ) was provided generously by Mandel Gail (State University of New York at Stony Brook). Leu positive blue colonies were isolated on His- Trp- Leu- drop out plates + X-gal. False positives were eliminated and clones that were reconfirmed in a second screen were selected and retested.

### Pull-down assay

Fusion proteins were expressed and induced according to standard protocols. The cultures were pelleted, resuspended in lysis buffer B (50 mM NaH_2_PO_4_, 750 mM NaCl, 20 mM Imidazole, and 1% Triton X 100), and sonicated for five pulses of 30 s each. The bacterial protein extract containing the 6xHis-Hsp90α (120 μg) or 6xHis-ScRad51 (80 μg) bait proteins and 300 μl of Wash Buffer were added to 30 μl Ni-NTA resin particles. The samples were incubated for 1.5 h at 4°C on a shaker. Ni-NTA resin particles were washed four times with 200 μl of Wash Buffer (50 mM NaH_2_PO_4_, 300 mM NaCl, and 20 mM Imidazole, pH 8.0) and resuspended with 30 μl of Wash Buffer. Fifteen microliters of particles were transferred to new tube. The prepared bait 6xHis-Hsp90α or 6xHis-Rad51_Sc_/Ni-NTA resin particles were resuspended in 185 μl of Wash Buffer. One hundred microliters of the nuclear extracts were added and shaken for 2 h. The particles were washed four times with 200 μl Wash Buffer and once with 100 μl Elution Buffer (pH 8.3). Twenty microliters of Laemmli sample buffer were added to the particles, and then the sample was boiled at 95°C for five minutes and analyzed by SDS-PAGE.

### Total, cytoplasmic, and nuclear extracts and Western blotting

To obtain total extracts after the differentiation treatment, the cell monolayer was washed with phosphate-buffered saline (PBS) and suspended in an ice-cold lysis buffer-I containing Tris (50 mM, pH 7.9), NaCl (100 mM), urea (8 M), Triton X-100 (1%), glycerol (5%), SDS (0.1%), Nonidet P-40 (0.5%), EDTA (0.1 mM), EGTA (2 mM), NaF (50 mM), Na3VO4 (1 mM), phenylmethylsulfonyl fluoride (1 mM), DTT (0.1 mM), SDS (0.2%), and a protease inhibitor cocktail (Sigma-Aldrich, P8340); and incubated on ice for 10 min. Then, cells were collected by scraping, sonicated, and centrifuged at 18300 x g for 15 min at 4°C. To isolate cytosolic and nuclear extracts, cells transiently transfected at 15 h were washed twice with cold PBS 1X. The cell monolayer was harvested; lysed in an ice-cold lysis buffer-II containing Tris (20 mM, pH 8.0), NaCl (15 mM), KCl (60 mM), EGTA (0.5 mM), sucrose (0.3 M), Nonidet P-40 (0.25%), 2-mercaptoethanol (0.5 mM), phenylmethylsulfonyl fluoride (1 mM), and a protease inhibitor cocktail; and shaken for 15 min at 4°C. Then, cells were centrifuged at 1,800 × g for 10 min at 4°C. The supernatant was saved as the cytosolic fraction, and the pellet was suspended in ice-cold radioimmunoprecipitation assay (RIPA) buffer containing Tris (50 mM, pH 7.4), NaCl (150 Mm), SDS (0.1%), Nonidet P-40 (0.5%), EDTA (1 mM), EGTA (1 mM), NaF (1 mM), phenylmethylsulfonyl fluoride (1 mM), sodium deoxycholate (0.5%), and a protease inhibitor cocktail and was then sonicated and centrifuged at 18,300 x g for 15 min at 4°C. The supernatant was saved as the nuclear fraction. For the Western blot analysis, protein extracts (40, 80, or 100 μg) were separated in 5% or 8% SDS-PAGE gel and then transferred onto nitrocellulose membranes. The membranes were blocked overnight with 10% non-fat dry milk in TBST at 4°C. The membranes were then incubated with rabbit anti-REST (Millipore, 07–579), goat anti-Hsp90α (Santa Cruz Biotechnology, sc-8262), goat anti-Htt (Santa Cruz Biotechnology, sc-8767 or sc-8768), rabbit anti-PARP1 (Santa Cruz Biotechnology, sc-7150), mouse anti-GAPDH (Santa Cruz Biotechnology, sc-32233), rabbit anti-NSE (Santa Cruz Biotechnology, sc-15343), mouse anti-tubulin β3 (Bio Legend Inc, 801201), rabbit anti-lamin A/C (Santa Cruz Biotechnology, sc-H-110), mouse anti-NeuN (Millipore, #MAB377) or monoclonal anti-β-actin for 2 h at room temperature. After washing, membranes were incubated with horseradish peroxidase-conjugated secondary antibodies (1:500, 1:1000, or 1:5000) for 1 h at room temperature. Bands containing the proteins were visualized on x-ray film (Kodak) using an enhanced chemiluminescence (ECL) kit. A densitometric analysis of Western blot bands was performed using the software Image StudioTM Lite Ver 4.0. Signals were normalized to those of β-actin.

### Co-immunoprecipitation analysis

To determine the interaction between nHtt or mHtt with Hsp90, lysates from differentiated SH-SY5Y cells were transiently transfected with expression vectors of green fluorescent proteins (GFPs), GFP-480-68Q or GFP-480-17Q (control) for 15 h. Cells were prepared in lysis buffer and subjected to immunoprecipitation (IP) with anti-Hsp90α antibody (Abcam, AB133491) or anti-HA antibody (Sigma, H9658) as a control; then, the membrane was blotted with Htt antibody. To determine whether Hsp90α is associated with REST, differentiated SH-SY5Y cells were prepared in a lysis buffer containing HEPES (20 mM, pH 7.6), NaCl (150 mM), glycerol (2.5%), Nonidet P-40 (1%), EDTA (13.4 mM), EGTA (2 mM), NaF (50 mM), Na3VO4 (1mM), phenylmethylsulfonyl fluoride (1 mM), and a protease inhibitor cocktail. Lysates were subjected to immunoprecipitation with 2.5 μg of anti-REST, anti-Hsp90α, or nonimmune anti-HA antibody for 20 h at 4°C. Cells were then added with protein G (Invitrogen, 15920–010), incubated for 4 h, washed twice with lysis buffer without Nonidet P-40, and eluted with SDS-PAGE sample loading buffer. Immunoprecipitates were subjected to SDS-PAGE analysis followed by Western blot analysis with anti-Htt, anti-REST, or anti-Hsp90.

### Antisense oligonucleotides of HSP90

Antisense oligonucleotides of HSP90 are single-stranded 17-nt (Hsp90 ASO) was analyzed using the basic local alignment search tool (BLAST) based on Megablast general algorithm parameters. Differentiated SH-SY5Y cells were transfected with different concentrations of Hsp90 ASO using Lipofectamine 2000 (Invitrogen, P/N 52887) according to the manufacturer’s protocol. At 24, 48, and 72 h after transfection, cells were used for cell viability assays or harvested for analysis by SDS-PAGE. Differentiated SH-SY5Y cells were transiently co-transfected with expression vector GFP-68Q and 12 μM Hsp90 ASO or 12 μM Hsp90 mutant oligonucleotide (control) using Lipofectamine 2000 (Invitrogen) according to the manufacturer’s protocol. At 24, 48, or 72 h after transfection, cells were used for cell death assays or immunofluorescence or were harvested for analysis by SDS-PAGE. All oligonucleotides were purchased from Sigma, and their sequences are shown in S1 Table.

### Plasmid and transient transfection

The wild-type and mutant full-length Htt expression vectors GFP-C2-Htt 480-17Q-HA and GFP-C2-Htt 480-68Q, express the green fluorescent protein and Huntingtin with 17 and 68 glutamines respectively, and were kind gift of Dr. Humbert S. (Institute Curie-UMR 146 du CNRS, Centre Universities Orsay, France). For convenience, when we mention in the text, 17Q or 68Q, we refer to the plasmids GFP-Htt-480-17Q, and GFP-Htt-480-68Q respectively. Differentiated SH-SY5Y cells were transiently transfected with 17Q or 68Q vectors using Lipofectamine 2000 (Invitrogen) according to the manufacturer’s protocol. At 6 h post-transfection, cells were treated with 1 μM GA (Sigma, G3381) or 50 nM PU-H71 (TOCRIS bioscience, 3104) for 24 h. In addition, the analysis of the expression of the Huntingtin protein is carried out by fluorescence of the green protein.

### Cell viability assay

SHSY5Y cells were plated on 96 well plates (Costar) at a density of 13000 cells per well of 100-μl of medium and were then differentiated. After the treatment periods, cell media were replaced with DMEM containing 10% 3-(4, 5-dimethylthiazol-2-yl)-2,5-diphenyltetrazolium bromide (MTT) (Sigma) for 3 h at 37°C. Then, all remaining media and MTT solutions were collected, and the resultant formazan crystals were solubilized in 100 μl of MTT solubilization solution (Sigma) and shaken for 10 min. Absorbance was measured at 570 nm with an ELISA Reader (SynergyH4 Hybrid Reader, BioTek). Assay values obtained upon vehicle treatment were set as 100%, and complete inhibition of MTT reduction (0%) was defined as the value obtained following the addition of the MTT solubilization solution. Cell death was determined by the following formula: % of cell death = 100% − % of cell viability.

### Confocal microscopy/immunofluorescence

SH-SY5Y cells were grown and differentiated on collagen coated coverslips, and were transient transfected with vectors GFP-17Q or GFP-68Q. At 24 h, cells were washed with PBS, fixed with 3.5% paraformaldehyde in PBS for 20 min at room temperature, permeabilized with 0.2% Triton X-100 for 10 min, and then blocked with 0.5% gelatin and 0.5% BSA for 20 min at 37°C. Fixed cells were then incubated overnight in primary antibody rabbit anti-REST at 4°C, washed with PBS, and incubated with TRITC (Vector Laboratories, INC. DI-1094) conjugated secondary antibody for 1 h at 37°C. For the immunodetection of neuronal markers, we used antibodies specific for the NeuN, β3 tubulin and NSE (Neuron-Specific Enolase). The *NeuN* gene has its highest expression in the central nervous system and plays a prominent role in the regulation of adult brain function. β3 tubulin, is primarily expressed in neurons and may be involved in neurogenesis and axon guidance, and NSE (Neuron-Specific Enolase) is found in mature neurons.

To determine the interaction between Htt or mHtt with Hsp90, SH-SY5Y cells were grown and differentiated on collagen coated coverslips, and were transient transfected with vectors GFP-17Q or GFP-68Q. At 24 h, were incubated overnight in primary antibody mouse anti-Hsp90 at 4°C, washed with PBS, and incubated with Texas Red (Vector Laboratories, INC. TI-2000) conjugated secondary antibody anti-mouse for 1 h at 37°C. To determine the endogenous interaction between REST with Hsp90, SH-SY5Y cells differentiated were incubated overnight in primary antibody rabbit anti-REST and mouse anti-Hsp90 at 4°C, washed with PBS, and incubated with Alexa Fluor 488 (Invitrogen, A21206) conjugated secondary antibody anti-rabbit and Texas Red (Vector Laboratories, INC. TI-2000) conjugated secondary antibody anti-mouse for 1 h at 37°C. These cells were subsequently washed and counterstained with DAPI for 15 minutes and washed with PBS. Coverslips were mounted on glass slides. Fluorescence was observed using confocal laser scanning microscopy (LEICA, DMI4000B) using a 63x oil immersion objective.

### Statistical analysis

The results are independently expressed as means ± SEMs for at least three of the experiments. For statistical comparisons, quantitative data were analyzed by one-way or two-way analysis of variance (ANOVA) and Dunnett’s post-test in GraphPad Prims version 5.00 for Windows (GraphPad Software; San Diego, California, USA; www.graphpad.com). P-values < 0.05 was considered significant.

## Results

### REST interacts with Hsp90

**Fig 1:**
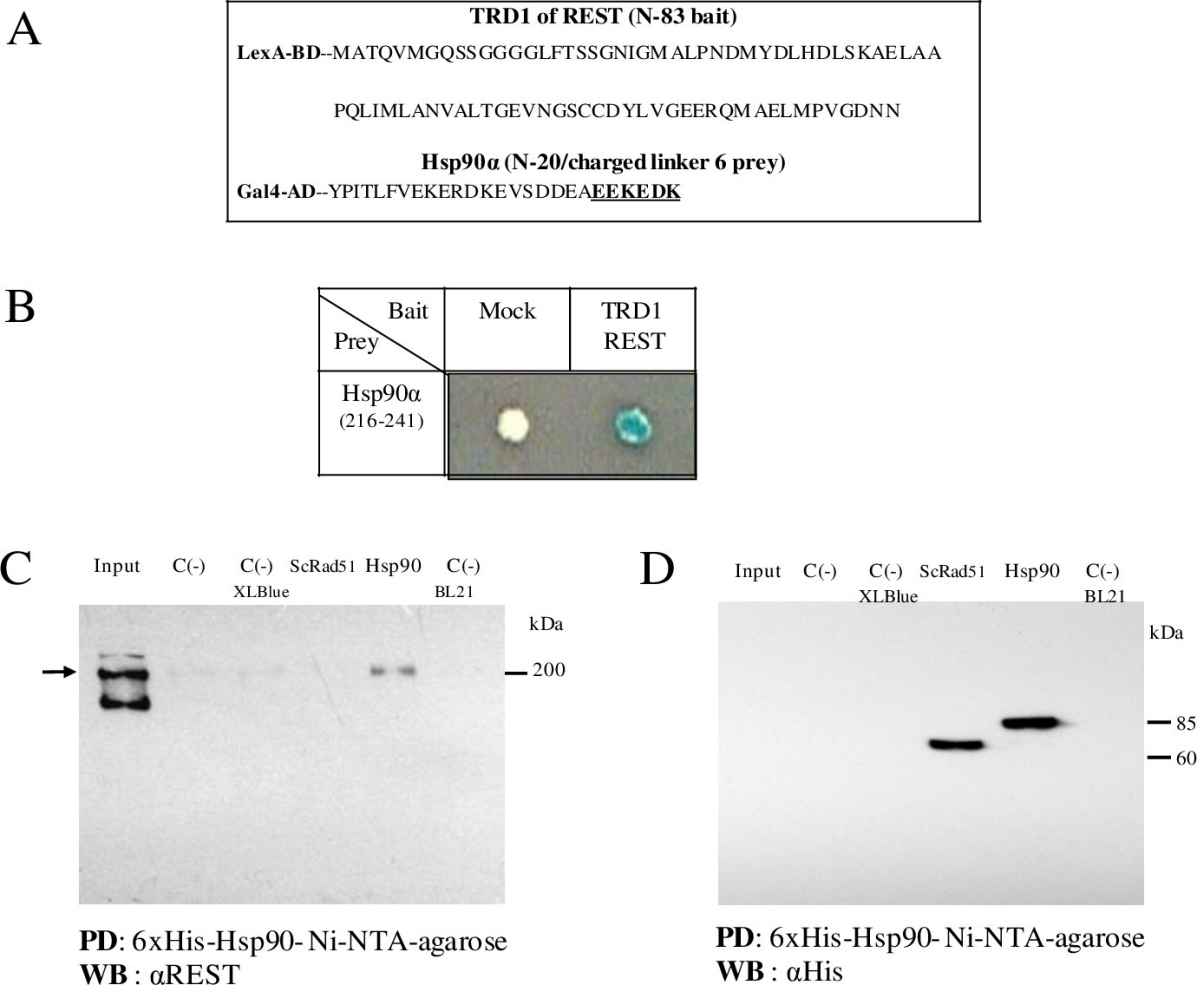
Yeast two-hybrid screen shows that the N-terminus of Hsp90α interacts with TRD1 of REST. *(A)* Sequence of TRD1 (N- termini of REST, 1–83 aa), and Hsp90α N- termini fragment [216–241 aa, including the highly charged linker (236–241 aa indicated in bold and underlined)]. (*B)* A representative Hsp90α prey plasmid (clone 17.52-SK) was transformed into L40 yeast expressing REST bait or empty plasmid (negative control). Transformed yeast were grown on His- /Trp- /Leu- drop out plates + β -X-gal. 17.52-SK is a clone identified through library screening that contained part of the human *HSP90* gene (*HS90AA1*). (*C)* His-tag pull-down assays were performed using nuclear protein extracts from synchronized HeLa cells. These extracts were incubated with purified 6xHis-Hsp90α protein bound to Ni-NTA agarose beads (lane 5). Nuclear protein extracts from synchronized HeLa cells were used as Input and as a negative control to rule out non-specific binding by Ni-NTA-agarose (lane 2). Additional negative controls used were bacterial extract (BL21 or XL Blue, lane 3 and 6) and 6xHis-ScRad51 (lane 4, Sc: *S*. *cerevisiae*). The autoradiogram shows bound protein after elution from Ni-NTA agarose beads and separation by 8% SDS-PAGE, followed by immunoblotting with anti-REST antibody (Millipore-Upstate 07–579). The molecular weights are indicated to the right of panels C and D (kDa). The arrow indicates the position of the REST endogenous dimer. (*D)* Immunoblotting of a His-tag pull-down assay using anti-His tag antibody. PD: pull-down. WB: western blot.

In neuronal cells, Htt interacts with REST and maintains it in the cytoplasm, reducing its availability to RE1-binding sites and ultimately allowing gene transcription [6]. Loss of this activity in mHtt may contribute to disease pathogenesis in HD [33, 34]. Given the importance of REST in this disease, little is known about its stability. In an attempt to identify other proteins capable of interacting with REST, we carried out an assay with the yeast double hybrid system, this system is physiologically adequate to analyze interactions between proteins, maintaining its conformation. In this way we identified several interacting proteins, however the protein that was relevant for our purpose, turned out to be the Hsp90 protein, and this interaction was corroborate by His-tag pull down assays (Fig 1). Hsp90 is a chaperone involved in the maturation, stability and degradation of proteins. In previous results the interactomic analysis of REST find different proteins including link with the transcriptional suppressor TRIM28 and other 240 proteins, but does not find the Hsp90 [35].

### SH-SY5Y cells differentiation and mHtt

**Fig 2:**
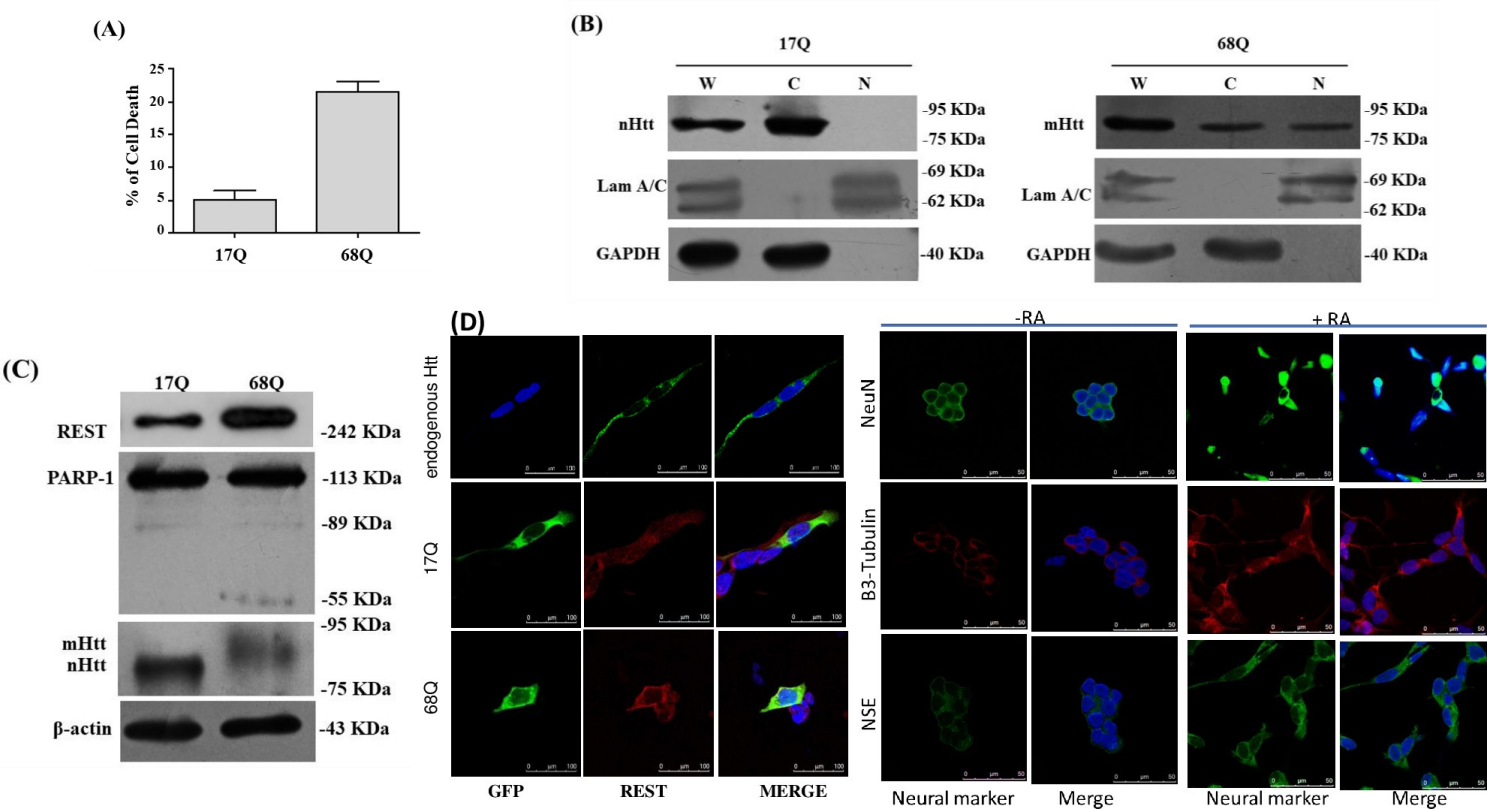
Transfected mHtt increases the level of REST and induces apoptosis in differentiated SH-SY5Y cells. (A) Cell death of differentiated SH-SY5Y cells transiently transfected with expression vectors GFP-480-17Q or GFP-480-68Q was determined by the MTT method 24 h after transfection. The 0% values of viable cells were defined by the measurement of cells transfected with expression vector for GFP. At 24 h, mHtt resulted in more death compared with wild-type Htt. Data are representative of three independent experiments (*P*<0.005). (B) Analysis of the subcellular localization of mHtt. Differentiated SH-SY5Y cells transiently transfected at 15 h were fractionated into cytoplasmic (C) and nuclear (N) fractions and were analyzed by Western blot for expression of normal and mutant Htt, respectively. Whole cell extract (W) served as a control. GAPDH and Lamin A/C served as a loading control for cytoplasmic and nuclear proteins, respectively. (C) PARP proteolysis was measured in lysates from cells transfected with expression vectors. Immunoblotting showed that mHtt is toxic and induces more cleavage PARP-1 products than normal Htt. Also, mHtt increased REST expression by around double. β-actin was used to assess the quality of total protein loading. *P*<0.005. (D) Confocal microscopy of cells transfected with expression vectors at 24 h. GFP fluorescence (green), REST is in red and DNA stained with 4’ 6-diamido-2-phenylindole (DAPI) (blue). Cells tranfected with expression vector GFP-480-68Q showed signs of neurodegeneration and early apoptosis events such as morphological changes, membrane blebbing, cell shrinkage, shorter neurites, and sometimes fragmented nuclei, especially condensed and/or fragmented nuclei compared with normal Htt. As controls for neuronal differentiation, we include three neuronal markers; NeuN, β3-Tubulin and NSE (Neuron-Specific Enolase).

The characterization of the Huntington disease cell model, and the effect of the mutant protein of huntingtin (mHtt), was carried out in the SKSH5Y cell line, inducing its differentiation with retinoic acid, and transfection of the construct, which contains the expansion of the repeated CGT. During differentiation, SH-SY5Y cells stopped proliferation and underwent morphological changes; these cells developed mature neurites (S1A Fig) and showed a significant increase in neuron-specific markers, e.g. NSE (Neuron-Specific Enolase), NeuN and β3-tubulin, which were expressed in both undifferentiated (-RA) and differentiated cells (+RA) (Fig 2D Right Panel +RA), and as shown in the representative Western blots in S1B Fig. Densitometry analysis showed that differentiated cells doubly overexpressed neuron-specific markers compared to undifferentiated cells, which were evident at 4 days of differentiation (S1C Fig). We explored whether mHtt induces neuronal-programmed cell death through apoptosis in differentiated SH-SY5Y cells. These cells were transiently transfected with expression vector GFP-68Q (expressing the HD exon 1 protein with 68 glutamines) or GFP-17Q (expressing the HD exon 1 protein with 17 glutamines) and were analyzed 24 h after transfection. Fig 2A shows that neurons transfected with Htt-68Q showed a higher cell death rate than those transfected with Htt-17Q (P<0.005). Additionally we analyzed the subcellular localization of mHtt; transiently transfected cells were harvested after 15 h, and a subcellular fractionation experiment was performed. The protein extract was analyzed by SDS-PAGE, and Western blot analyses were carried out. We detected mHtt protein (68Q) in cytoplasmic and nuclear fractions, whereas normal Htt protein (17Q) was only detected in the cytoplasm. The fractionation procedure efficiency was verified by analyzing the distribution of Laminin A/C, a nuclear protein, and the cytoplasmic protein GAPDH (Fig 2B). Cell dehydration occurs during the early stages of apoptosis. The loss of intracellular water results in changes in cell shape and size, as cells elongate and diminish in size. In addition, chromatin condensation starts at the nuclear periphery. At later stages of apoptosis, nuclear fragmentation occurs [36]. Expression of N-Htt-Q68 has been reported to cause the induction of apoptosis in undifferentiated SH-SY5Y cells [37], and mHtt induces a cell type-specific neurodegeneration similar to that seen in HD in vivo [3]. Our results coincide with the previously reported results and give us confidence in the model we use. In addition mHtt induces apoptotic events, we measured poly (ADP-ribose) polymerase-1 (PARP-1) cleavage products in Western blot analyses. Cells transiently transfected with vector Htt-68Q contained more cleaved PARP-1 (55 KD) and showed greater REST expression than cells transiently transfected with vector Htt-68Q (Fig 2C) (*P*<0.05). Using confocal microscopy and staining with DAPI, we observed early apoptosis events only in cells transfected with mHtt after 24 h, including morphological changes, membrane blebbing, cell shrinkage, shorter neurites, and fragmented nuclei. Moreover, we observed that HTT-68Q formed aggregated proteins (Fig 2D). To validate our neuronal cell model, we differentiated the cells with retinoic acid, as described in Material and methods, and we used three neuron-specific markers; NeuN, beta III tubulin and NSE. We observe cells that acquire the neuronal phenotype in the presence of retinoic acid and express neuronal markers, as indicated in Fig 2D on the right. In contrast, cells not treated with retinoic acid do not develop the neuronal phenotype and express basal levels of neuronal markers. We demonstrate that terminally differentiated SH-SY5Y cells express neuronal proteins with a localization that is indicative of mature neurons. NeuN, this gene has its highest expression in the central nervous system and plays a prominent role in the regulation of adult brain function. β3 tubulin, is primarily expressed in neurons and may be involved in neurogenesis and axon guidance, and NSE (Neuron-Specific Enolase) is found in mature neurons.

### REST is an Hsp90 target protein

**Fig 3:**
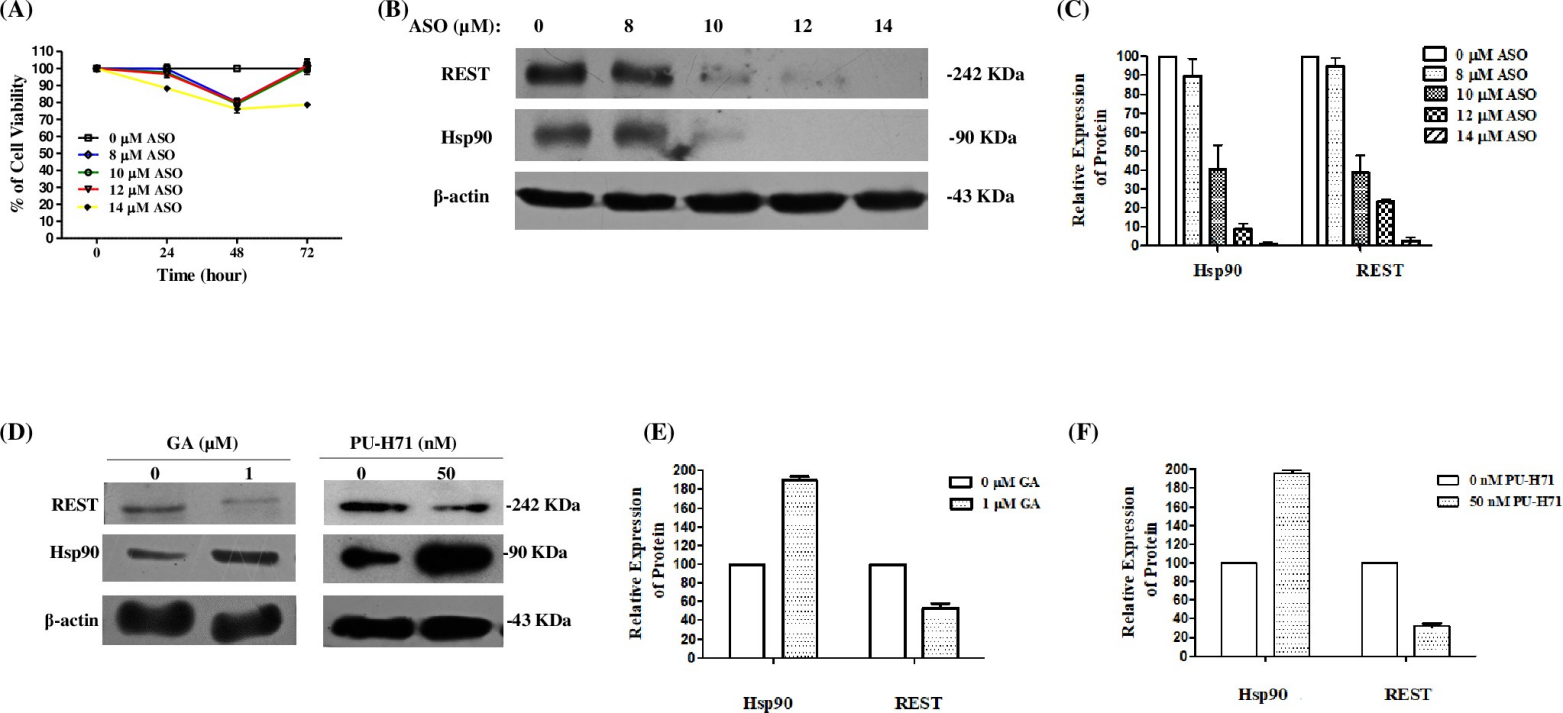
The decrease or inhibition of Hsp90 induced REST degradation. (A) Effect of Hsp90α ASO on cell viability. Differentiated SH-SY5Y cells were transfected with different concentrations of Hsp90α ASO. Cell viability was estimated after different periods (hours) by the MTT method. The viable cells’ 100% value was defined based on that obtained for non-transfected cells (*P*<0.0001). (B) Western blot analysis showed that the levels of REST and Hsp90α dramatically reduced in a dose-dependent manner at 48 h because of Hsp90α ASO. β-actin was used as a protein loading control. (C) The densitometry analysis of bands representing means ± SDs of three independent experiments (*P*<0.05). (D) The level of REST dramatically reduced in differentiated SH-SY5Y cells treated with GA (1 μM) or PU-H71 (50 nM) at 24 h. (E) The REST level decreased by GA more than 50% and (F) PU-H71 more than 80%, respectively. The presented results correspond with three separate experiments.

To investigate the possible effects that the inhibition of the expression of Hsp90, by an antisense oligonucleotide (ASO), could have on REST levels, a Knowdown was performed. We first determined the optimal concentration of the antisense oligo and then differentiated cells were transfected. Differentiated SH-SY5Y cells were transfected with Hsp90-ASO at concentrations ranging from 8 to 14 μM, and cell viability was then estimated at 24, 48, and 72 h by MTT assays. As shown in Fig 3A, all concentrations had no significant effect on cell viability after 24 h; however, cell viability decreased 20% after 48 hours (P<0.0001). At 72 h, the differences were not statistically significant. Western blot analysis showed that Hsp90-ASO had an inhibitory effect on Hsp90 protein expression and REST levels. Both were dramatically down-regulated in a dose-dependent manner by Hsp90 ASO (Fig 3B); proteins levels decreased by more than 80% (*P*<0.05) after 48 h of transfection (Fig 3C).

In addition to inhibiting the expression of Hsp90 with antisense oligos, we decided to evaluate whether specific inhibitors of the activity of the chaperone Hsp90 has a similar effect. For it we decided to standardize the conditions in our cellular model, briefly: differentiated SH-SY5Y cells were treated with different concentrations of the Hsp90 inhibitors geldanamycin (0, 0.5, 1, and 2 μM) and PU-H71 (0, 50, 100, and 200 mM), and effects on cell viability were analyzed at 24 h. Cell viability decreased in a dose-dependent manner (*P*<0.05) (S1A Fig). Hsp90 level and REST stability were also measured at 24 h (S2B and S2C Fig). These inhibitors specifically bind to the N-terminal ATP-binding site of HSP90, destabilizing the association between HSP90 and its client proteins [38–40].

**Fig 4:**
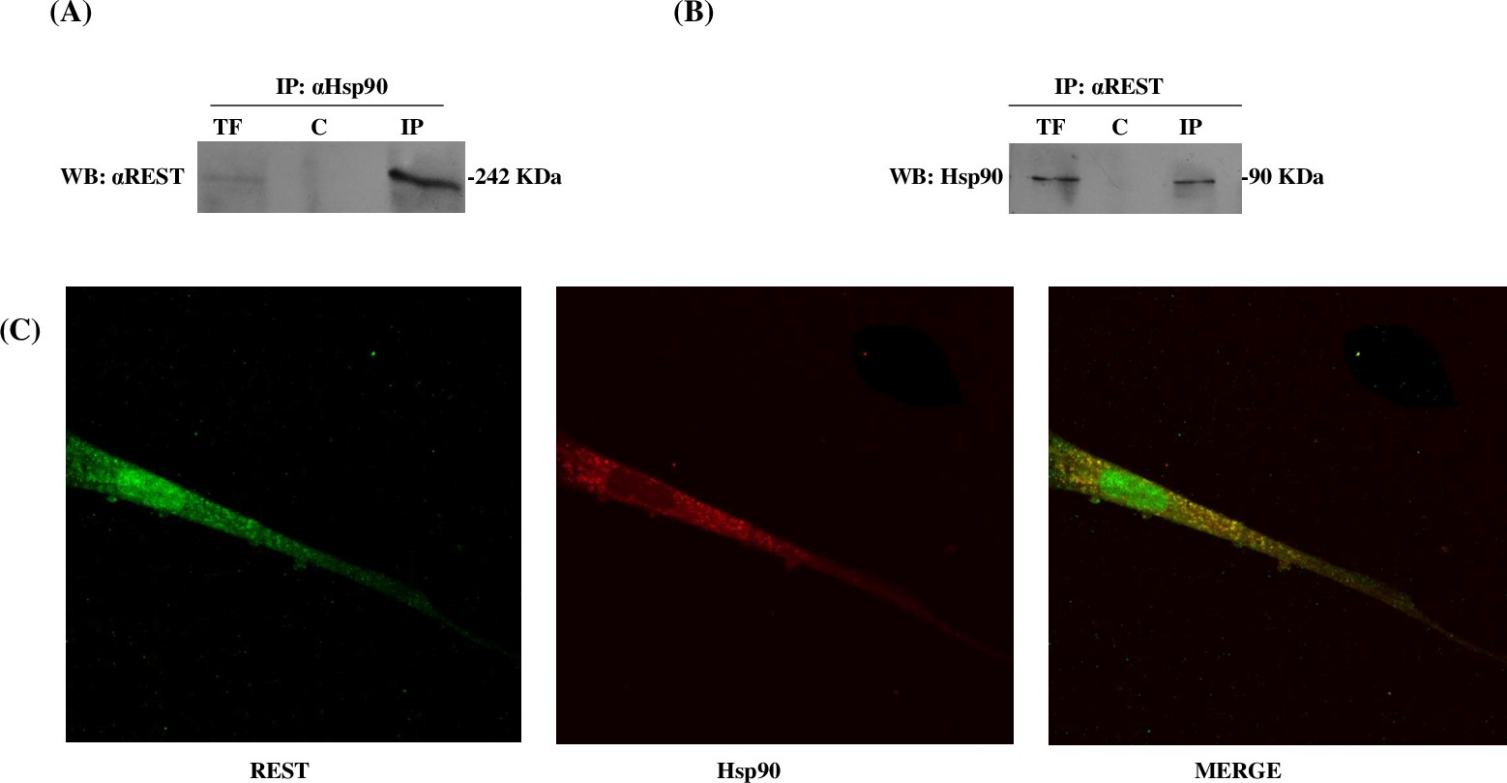
Analysis of the interaction between Hsp90 and REST. (A) Differentiated SH-SY5Y cell lysates were prepared in lysis buffer and subjected to immunoprecipitation (*IP*) with anti-Hsp90 antibody or anti-HA antibody as a control (c). After SDS-PAGE and blotting, the membrane was probed with REST antibody. (B) Differentiated SH-SY5Y cell lysates were prepared in lysis buffer and subjected to immunoprecipitation (*IP*) with anti-REST antibody or anti-HA antibody as a control (C). Then, the membrane was blotted with Hsp90 antibody. (C) Differentiated SH-SY5Y cells, were doubly stained for Hsp90 (in red) and REST (in green); images were captured by confocal laser microscopy. REST is visualized in green and Hsp90 in red.

Once the conditions were standardized, we proceeded to evaluate the effects of the inhibitors on the expression of REST. Differentiated SH-SY5Y cells were treated with the Hsp90 inhibitors GA (1μM) or PU-H71 (50 nM) at 24 h. The Western blot in Fig 3D shows that both GA and PU-H71 decreased Hsp90 levels by more than 80% (*P*<0.05); specifically, GA decreased REST by more than 50% and PU-H71 by more than 70% (*P*<0.05) (Fig 3E). Likewise, the profiles for REST and Hsp90 were time dependent. Then, to explore whether endogenous Hsp90 was associated with endogenous REST in differentiated SH-SY5Y cells, we carried out co-immunoprecipitation experiments with anti-REST and anti-Hsp90 antibodies, and the precipitates were analyzed with the same pair of antibodies. The immunoblot showed that REST is HSP90-associated protein (Fig 4A and 4B). Next, we examined this interaction by confocal microscopy. The merged staining patterns of Hsp90 and REST showed co-localization (see Fig 4C).

Together all these results show that the synthesis and activity of Hsp90 are important to maintain the stability of REST, and may be a potential target for diseases where the overexpression and /or localization of the neuronal repressor REST plays an important role.

### Genetic or pharmacological Hsp90 inhibition induces mHtt degradation

**Fig 5:**
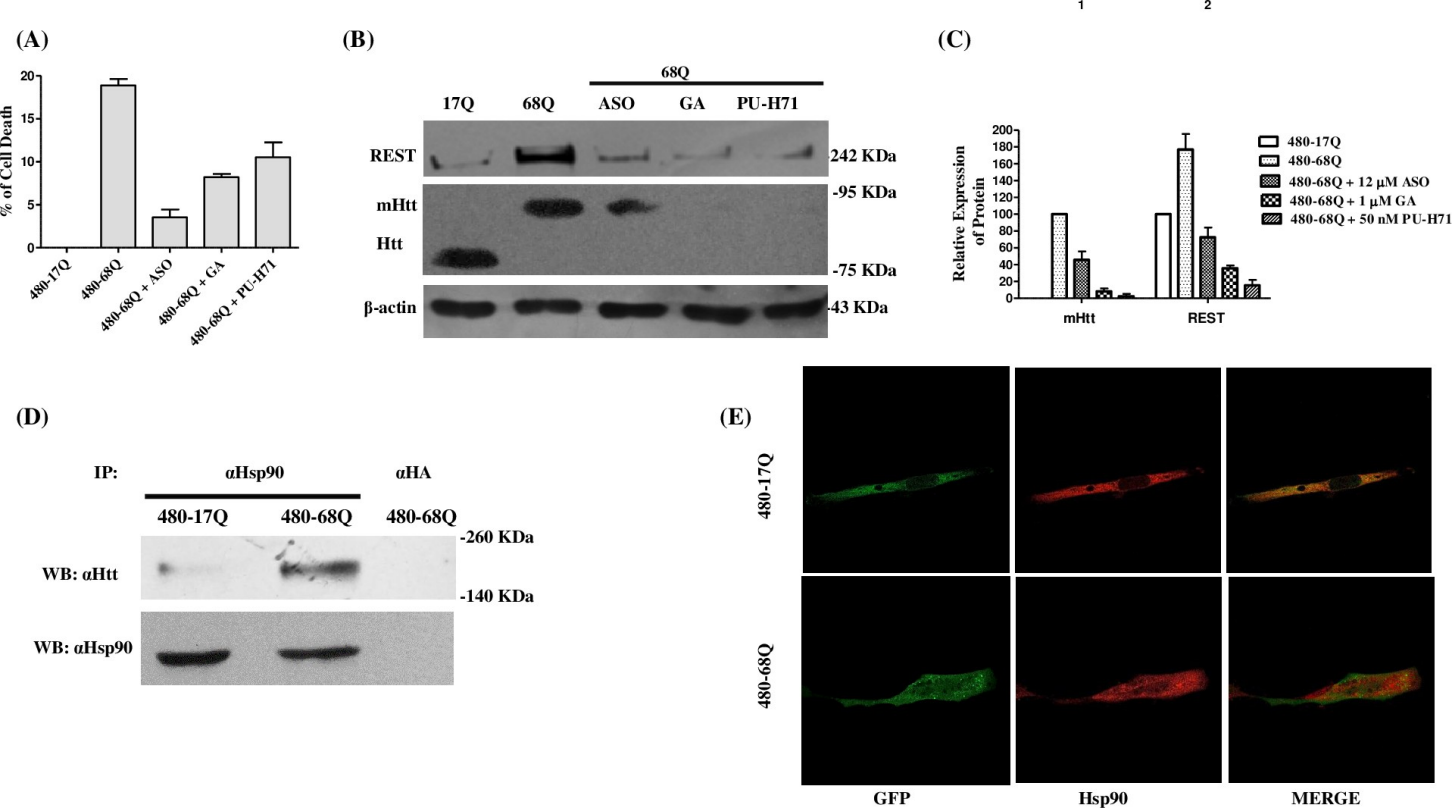
Inhibition of Hsp90 induces mutant huntingtin degradation. (A) Differentiated SH-SY5Y cells transiently transfected with expression vector GFP-480-68Q were treated at 6 h with GA (1 μM) or PU-H71 (50 nM) or were transiently co-transfected with vector GFP-480-Htt68Q and Hsp90 ASO (12 μM). After 24 h, cell death was analyzed. (B) Cells lysates were analyzed by Western blotting. (C) Protein levels were compared to those of control cells solely transfected with expression vector GFP-480-17Q. β-actin was used to assess total protein loading. (D) Differentiated SH-SY5Y cell lysates were prepared in lysis buffer at 15 h after their transfection with expression vector GFP-480-17Q or GFP-480-68Q and were subjected to immunoprecipitation (*IP*) with anti-Hsp90 antibody or anti-HA antibody as a control. Then, the membrane was blotted with anti-Htt antibody (upper), and the membrane was stripped and blotted with anti-Hsp90 (lower). (E) Protein interactions were analyzed by confocal microscopy. The staining of exogenous nHTT or mHtt (green) and Hsp90 (red) were merged. Hsp90 is observed co-localizing with mHtt.

As Hsp90 ASO and the Hsp90 inhibitors GA and PUH-71 were effective in decreasing REST levels. At following, we tested whether these inhibitors also effectively decrease mHtt levels. Differentiated SH-SY5Y cells transiently transfected with expression vector GFP-68Q were treated for 6 h with GA (1 μM) or PU-H71 (50 nM). In addition, differentiated SH-SY5Y cells were transiently co-transfected with vector GFP-68Q and Hsp90 ASO (12 μM). After 24 h, neurons co-transfected with Htt-68Q and Hsp90 ASO showed significantly less programmed cell death compared to those transfected with Htt-68Q (Fig 5A). Hsp90 inhibitors GA and PU-H71 also reduced programmed cell death (*P*<0.05). Cell lysates were separated by 8% SDS-PAGE and analyzed by Western blot (Fig 5B). Treatments with GA and PU-H71 significantly reduced the mHtt level (P<0.005) by more than 70% and 90%, respectively, as well as the REST level induced by mHtt. Hsp90 ASO efficiently decreased the mHtt expression by more than 50% as well as the REST level (P<0.005). The reduction in mHtt level was compared to that in control cells that were only transfected with expression vector GFP-68Q, and the level of REST was additionally compared with control cells solely transfected with expression vector GFP-17Q (Fig 5C). To confirm these results, transfected cells with expression vector GFP-68Q, were treated with GA (1 μM), PU-H71 (50 nM), or Hsp90 ASO (12 μM). After 24 h, cells were stained with DAPI and examined by confocal microscopy. These images are shown in S3 Fig, a population of at least 300 transfected cells was analyzed, and the image represents the average of them. The results show that the mHtt and REST levels decrease. As a step in determining whether endogenous Hsp90 is associated with exogenous nHtt or mHtt in differentiated SH-SY5Y cells, we performed co-immunoprecipitation experiments with anti-Hsp90 or anti-HA (control) antibodies. The precipitates were analyzed with anti-Htt antibody. Hsp90 had a binding preference for mHtt versus Htt (Fig 5D). Afterwards, we examined the Hsp90-mHtt interaction by confocal microscopy. The staining patterns of merged images corresponding to exogenous Htt or mHtt with Hsp90 (Fig 5E) showed that Hsp90 co-localized with mHtt.

### The Genetic inhibition of Hsp90 decreased cellular apoptosis

**Fig 6:**
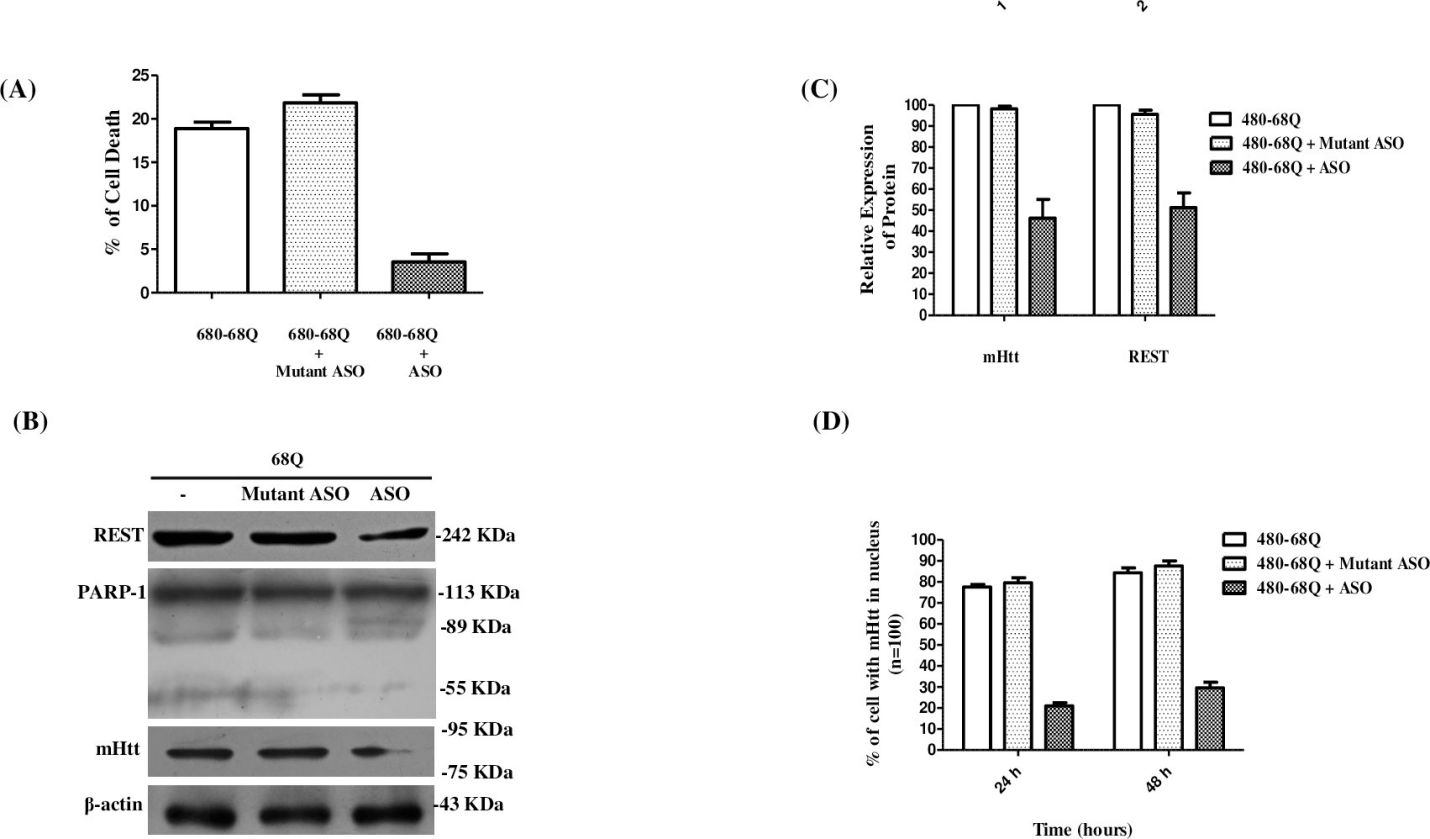
Inhibition of Hsp90 decreased cellular apoptosis. (A) Differentiated SH-SY5Y cells were transiently co-transfected with expression vector for GFP-68Q and 12 μM Hsp90 ASO or 12 μM Hsp90 mutant oligonucleotide. Their effects on cell viability were analyzed up to 48 h. Transfected cells with expression vector for GFP-480-17Q are normalized to 0% (*P*<0.05). (B) Cells lysates were analyzed by Western blotting, β-actin was used to assess total protein loading. (C) Protein levels were compared to control cells, which were only transfected with expression vector for GFP-68Q. (D) Quantitative analysis to determine the percentage of cells that presented to mHtt in nucleus. The results represent the mean and SEM (n = 100) from a series of three separate experiments (*P*<0.05).

As Hsp90 ASO was effective in decreasing the mHtt level. In addition, we tested whether Hsp90 ASO was effective in reducing programmed cell death and in rescuing cells from mHtt-induced apoptosis. Differentiated SH-SY5Y cells were transiently co-transfected with expression vector GFP-68Q and 12 μM Hsp90 ASO or 12 μM Hsp90 mutant oligonucleotide. Fig 6A shows that cells co-transfected with Htt-68Q and Hsp90 ASO showed less than 5% programmed cell death at 48 h. Meanwhile, neurons co-transfected with Htt-68Q and Hsp90 mutant oligonucleotides showed nearly 22% programmed cell death, surpassing the 20% programmed cell death of neurons co-transfected with expression vector GFP-68Q (*P*<0.05). Cell lysates obtained from the same experiments at 48 h, were analyzed by Western blot, as shown in Fig 6B. The lane corresponding with cells co-transfected with Hsp90 ASO contained less cleaved PARP-1 products (55KD); in addition, mHtt (P<0.005) and REST levels (P<0.005) significantly reduced by more than half compared to cells co-transfected with Hsp90 mutant oligonucleotide (Fig 6C). We examined whether Hsp90 ASO has an effect on the percentage of cells containing mHtt in nucleus, we found at 24 h and 48 h that the percentage of cells co-transfected with mHtt and Hsp90 ASO was of 21.1% and 29.66%, compared to without Hsp90 ASO that was of 77.66% and 84.33%. This effect was not observed with Hsp90 mutant ASO. Differences were not statistically significant between control and Hsp90 mutant ASO only with Hsp90 ASO (p<0.05) (6D).

**Fig 7:**
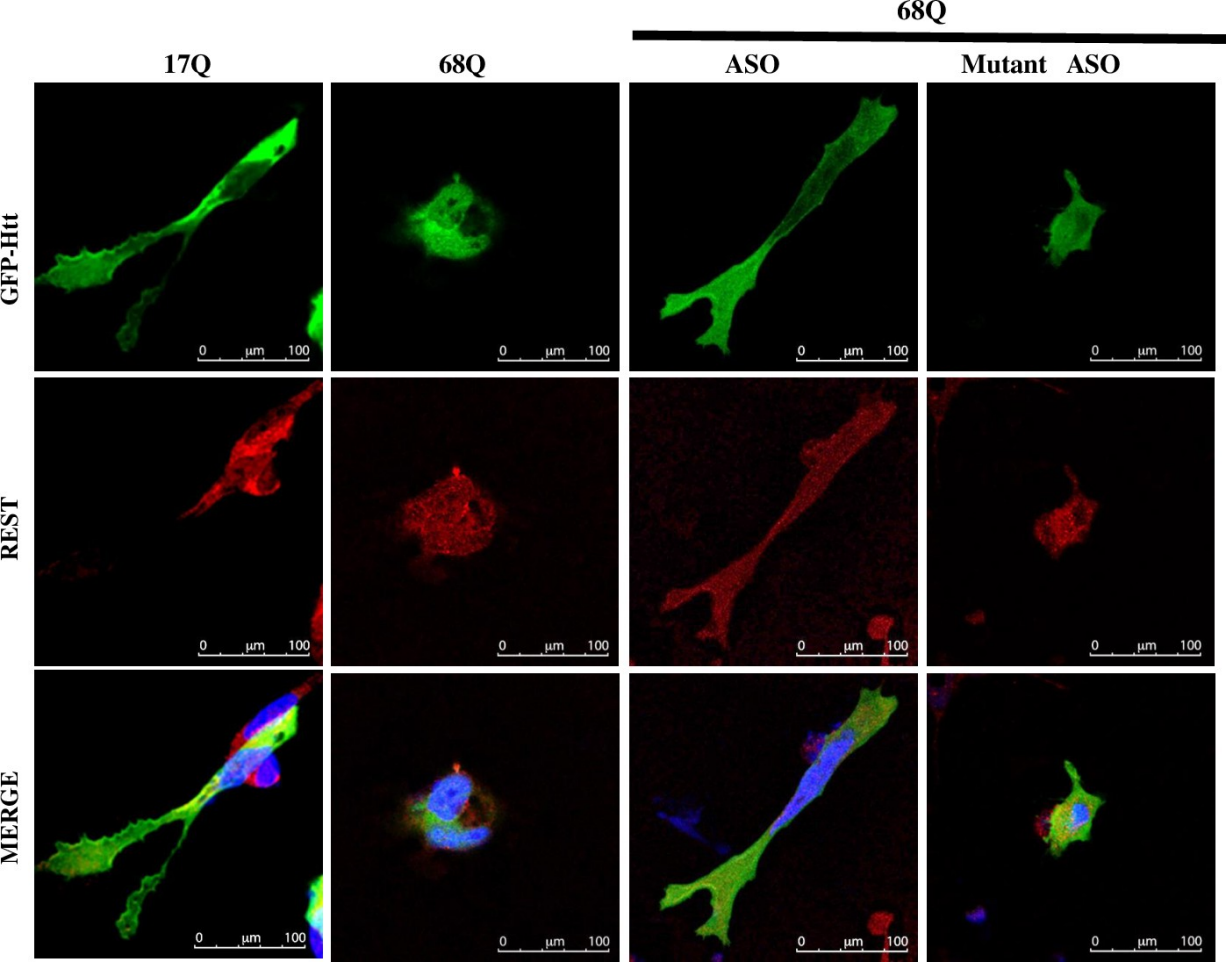
Inhibition of the expression or activity of Hsp90, rescues of the cell death to differentiated cells and transfected with mutant Htt. Differentiated SH-SY5Y cells transfected with expression vectors GFP-480-17Q or GFP-480-68Q (fluorescence green), for 24 h. REST in red and DNA stained DAPI (blue). Cells transfected with expression vector GFP-480-68Q or co-transfected with Hsp90 mutant ASO showed signs of neurodegeneration and early apoptosis events including morphological changes, membrane blebbing, cell shrinkage, shorter neurites, and sometimes more fragmented nuclei compared with GFP-480-17Q. Antisense oligos against Hsp90 (ASO) decreased the levels of mHtt and REST in the nucleus, and GA or PUH-71 presented similarly behavior; decreased mHtt and REST level, and they had protective effects against mHtt toxicity. A population of at least 300 co-transfected cells, with expression vector GFP-480-17Q or GFP-480-68Q and Hsp90-ASO, were analyzed and the image shown is representative of them.

To support these results, cells co-transfected with expression vector GFP-68Q and Hsp90 ASO or Hsp90 mutant ASO were stained with DAPI after 24 h and analyzed by confocal microscopy. The images are shown in Fig 7, we observed Htt only in the cytoplasm, and no morphological changes nor nuclear fragmentation were observed (line 17Q) and line (68Q plus ASO). In contrast, cells transfected with Htt-480-68Q (line 68Q) shown membrane blebbing and cell shrinkage. Moreover, the cells co-transfected with the Hsp90 mutant oligonucleotide showed early apoptosis events, including morphological changes, membrane blebbing, and cell shrinkage. Occasionally, nuclei were fragmented. Additionally, when the expression of mHtt and REST proteins, in the presence of the specific inhibitors of Hsp90, were analyzed, we find healthy cells with basal levels of mHtt and REST and the apoptosis process is absent (S3 Fig, lanes GA and PU-H71).

Therefore, Hsp90 ASO has a protective effect against mHtt toxicity, but Hsp90 mutant oligonucleotide showed no protection. These results indicate that in differentiated SH-SY5Y cells, Hsp90 ASO is highly effective in directly suppressing the HD gene and in significantly decreasing mHtt expression and REST stability.

Hsp90 belongs to the family of proteins called molecular chaperones that are involved in the stabilization and folding of many signaling proteins (client proteins), including key regulators of cell proliferation and survival such as receptor tyrosine kinases, metastable/mutant signaling proteins, transcription factors, and cell cycle regulators [38]. In neurodegenerative diseases, Hsp90 inhibition induces chaperones and is able to redirect neuronal aggregate formation. Therefore, it may exhibit protective potential against protein toxicity [39, 40].

We have already shown that Hsp90 prefers to bind to mHtt over nHtt, and that Hsp90-specific inhibitors GA and PU-H71 dramatically reduce mHtt stability. We have also demonstrated that Hsp90 ASO reduces Hsp90 expression and is highly effective in indirectly suppressing the HD gene. It significantly decreases REST and mHtt stability, reducing the percentage of cells with mHtt in the nucleus, as well as cell death and cytotoxicity in differentiated SH-SY5Y cells.

## Discussion

Several studies have clearly stated that REST is ubiquitously expressed during embryogenesis and that it plays a strategic role in the elaboration and specification of the neuronal phenotype by silencing a subset of neuron-specific genes in neural progenitors and non-neuronal cells [41]. In pluripotent stem cells and neuronal progenitors, REST actively represses a broad array of neuron-specific genes important to synaptic plasticity and structural remodeling, including synaptic vesicle proteins, neuroreceptors, and channels, in addition to microRNAs that regulate networks of non-neuronal genes. In neuronal progenitor cells, REST is regulated at the level of protein stability by the ubiquitin-proteasome system (b-TrCP) and deubiquitination activity (HAUSP) [42, 43], and the proteasomal degradation of REST is required during neuronal terminal differentiation for the cell to acquire its neuronal phenotype. The present study has demonstrated that terminally differentiated SH-SY5Y cells express neuronal proteins whose localization is characteristic of mature neurons. The *NeuN* gene is mostly expressed in the central nervous system, and it plays a prominent role in the regulation of adult brain function, while β3 tubulin is primarily expressed in neurons and may be involved in neurogenesis and axon guidance. For its part, neuron-specific enolase (NSE) is found in mature neurons. Our results show that the direct knockdown of endogenous Hsp90 results in a significant reduction of REST and mutant Huntingtin, reducing the percentage of cells presenting mHtt in the nuclei, and cells rescued from mHtt-induced cellular cytotoxicity. Additionally, Hsp90-specific inhibitors geldanamycin and PUH71 reduced mHtt and REST levels dramatically, thus providing neuroprotective activity.

Low levels of REST, which are achieved during differentiation, govern the expression of specific neuronal phenotypes; however, in adult neurons, REST levels are not always low, and they increase as a consequence of human aging [44]. However in Alzheimer´s disease REST is lost from the nucleus, and then it appears in the cytoplasm of autophagosomes, along with pathologically misfolded proteins [18].

In Huntington's disease, Htt regulates REST nuclear trafficking indirectly via the interaction of REST/NRSF-interacting LIM domain protein (RILP) with dynactin p150Glued*, an Htt-associated protein 1 (HAP1) that binds to the Htt protein. The formation of this complex causes REST retention in the cytoplasm, and oppositely, mHtt binding alters its conformation, resulting in the accumulation of REST in neuronal nuclei. The repression of neuronal genes containing RE1 sequences, including the brain-derived neurotrophic factor (BDNF) (21), is a direct effect of this erroneous REST transport.

The role of REST can be analyzed in different contexts: in the first scenario, where normal healthy aging conditions prevail, the level of REST is not always low; REST levels have been reported to increase progressively during aging in healthy humans, and nuclear translocation is beneficial because it can inhibit neuronal apoptosis and oxidative stress and also prevent the aggregation of amyloid-beta-protein, probably as a result of the upregulation of various protective genes and the downregulation of potentially toxic genes. In this scenario, REST is essential for maintaining neuronal viability, preserving cognitive capacity, and increasing neuronal longevity [44, 45].

In a neuropathological aging scenario, the loss of nuclear REST favors the aggregation of proteins and neurodegenerative diseases, such as Parkinson's and Alzheimer's. REST is reduced in stress conditions and in patients with Alzheimer's disease (AD), which suggests that increasing REST could be neuroprotective. REST is lost in mild cognitive impairment and Alzheimer's disease; chromatin immunoprecipitation analyses using deep sequencing (ChIP-seq) and expression analyses show that REST genes promote cell death and AD pathology, and induce the expression of stress response genes. Thus, the loss of nuclear REST in AD is associated with the epigenetic depression of potentially pathogenic genes; and a decrease in REST levels results in increased risk and impairment in AD [18, 19]. On the other hand, the loss of REST from the nuclei of dopaminergic neurons has been reported in patients with Parkinson's disease; instead the REST immunoreactivity was observed in Lewy bodies, apparently the accumulation of aggregates may prevent neuroprotective signaling through the REST sequestration mechanism [20]. By contrast, in Huntington's disease, the cytoplasm has been shown to have increased levels of mutant HD protein, leading to a decreased the REST sequestration capacity of the cytoplasm and allowing REST to enter the nucleus at higher than normal levels, enhancing its repressive effect and reducing the expression of neuronal survival factor BDNF.

## Conclusions

REST expression regulation and how it modulates the expression of its target genes should be understood as a dynamic process that depends on time, site, REST levels, and the coincidence of cofactors, corepressor expression, degradation enzymes, and stability. The present study analyzed the cross-talk between REST and Hsp90; if such cross-talk can be neutralized, it inhibits Hsp90 synthesis or activity.

In sum, the present paper reports for the first time on the association between REST and Hsp90. Hsp90 prefers to bind to mHtt over Htt, and our results show that REST protein levels can be reduced via the inhibition of Hsp90 expression with antisense oligos. Additionally, the present study demonstrates the possibility of reducing REST and mHtt expression using specific inhibitors of the chaperone's activity. Hsp90-specific inhibitors geldanamycin (GA) and PU-H71 inhibited the stability of mHtt and REST, providing excellent neuroprotective activity, and the inhibition of Hsp90 expression by Hsp90 ASO was found to decrease mHtt and REST levels, decreased the number of cells with mHtt in nuclei, and programmed cell death. Our data show that Hsp90 is necessary to maintain adequate levels of REST and mHtt, which suggests that the interaction between Hsp90-REST and Hsp90-Huntingtin could be a potential therapeutic target for treating Huntington's disease.

## Supporting information

S1 Table Sequences and position of Hsp90α oligonucleotides in *hsp*90AA1 mRNA. (TIF)

S1 Fig SH-SY5Y cells undergo morphological changes and express neuronal markers during RA-induced differentiation. Representative phase contrast images of (A) undifferentiated SH-SY5Y cells cultured in complete growth medium for 4 days and differentiated SH-SY5Y cells cultured with RA (10 μM) for 4 days in culture medium with 3% FBS. (B) Representative immunoblots of the neuronal markers neuro-specific enolase (NSE), NeuN and β3-tubulin. (C) Densitometry analysis of the bands representing means ± SDs of three independent experiments. β-actin was used as a loading control. *P*<0.05.
(TIF)

S2 Fig Effect of GA and PU-H71 on cell viability and REST stability. (A) Differentiated SH-SY5Y cells were treated for 24 h with increasing concentrations of geldanamycin (GA) or PU-H71. Their effects on cell viability were then analyzed. Cell viability decreased in a dose-dependent manner. Vehicle-treated cells were normalized to 100%. (*P*<0.05) (B) PARP cleavage products, Hsp90 level, and REST stability were determined by Western blot analyses at 24 h. β-actin was used to assess the quality of total protein loading that statistically differed from the corresponding control values. (C) The level of Hsp90 was decreased by GA and PU-H71. The REST level was dramatically down-regulated in a dose-dependent manner by GA and PU-H71. The levels were compared with the normal control (*P*<0.05). Densitometry analysis of bands represents the means ± SDs of three independent experiments. Drugs were assayed in triplicate. Errors bars show the SDs of the means.
(TIF)

S3 Fig Confocal microscopy of differentiated SH-SY5Y cells transfected with expression vectors for 24 h. GFP-Htt-480-17Q or GFP-Htt-480-68Q fluorescence (green), REST in red and DNA stained DAPI (blue). Cells transfected with expression vector 17Q or 68Q and treated with GA or PUH-71 presented similar cell protection results; decreased mHtt and REST level, and they had protective effects against mHtt toxicity, decreased mHtt in nucleus and REST level.
(TIF)
