# Supplementary figures and images for: The interaction between RE1-silencing transcription factor (REST) and heat shock protein 90 as new therapeutic target against Huntington’s disease
Source: PLoS One. 2019 Jul 30;14(7):e0220393. doi: 10.1371/journal.pone.0220393 (PMC6667143; doi:10.1371/journal.pone.0220393)

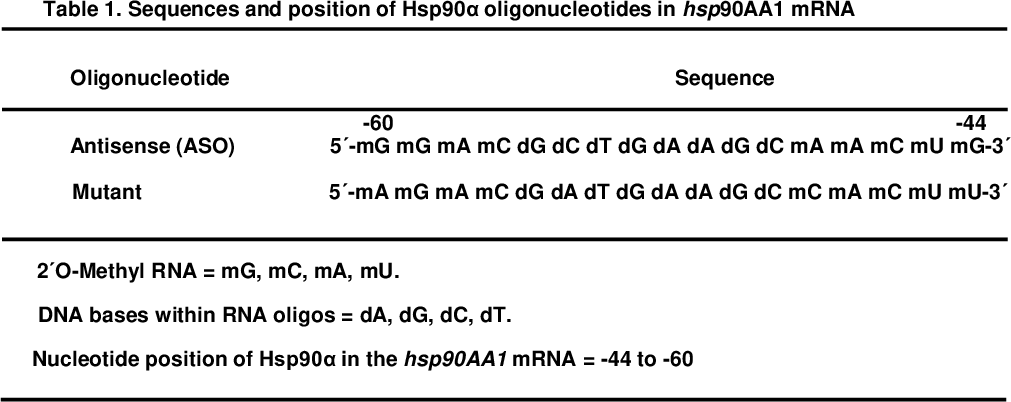

Supplement: S1 Table — (TIF) [file pone.0220393.s001.tif]

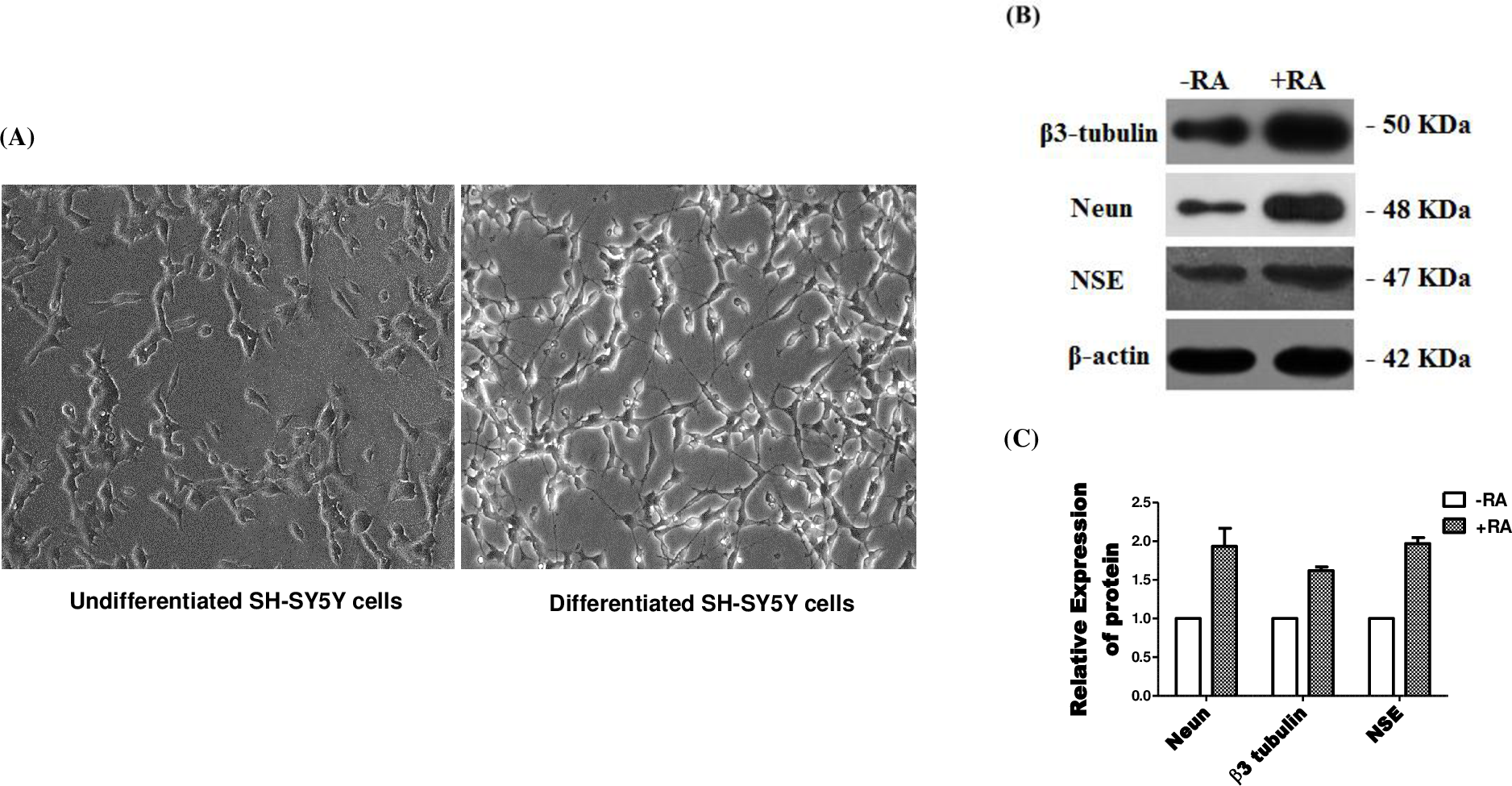

Supplement: S1 Fig — Representative phase contrast images of (A) undifferentiated SH-SY5Y cells cultured in complete growth medium for 4 days and differentiated SH-SY5Y cells cultured with RA (10 μM) for 4 days in culture medium with 3% FBS. (B) Representative immunoblots of the neuronal markers neuro-specific enolase (NSE), NeuN and β3-tubulin. (C) Densitometry analysis of the bands representing means ± SDs of three independent experiments. β-actin was used as a loading control. P<0.05. (TIF) [file pone.0220393.s002.tif]

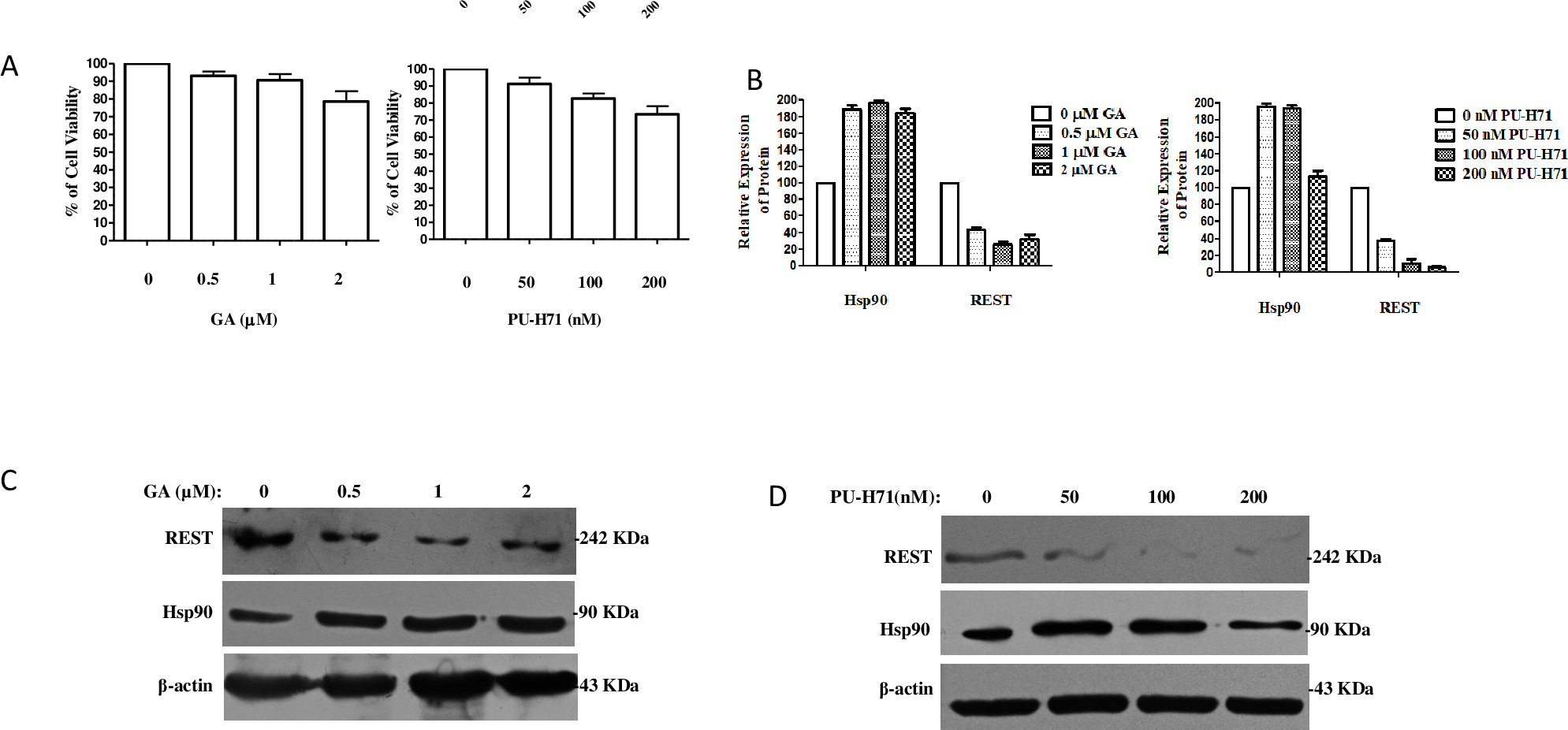

Supplement: S2 Fig — (A) Differentiated SH-SY5Y cells were treated for 24 h with increasing concentrations of geldanamycin (GA) or PU-H71. Their effects on cell viability were then analyzed. Cell viability decreased in a dose-dependent manner. Vehicle-treated cells were normalized to 100%. (P<0.05) (B) PARP cleavage products, Hsp90 level, and REST stability were determined by Western blot analyses at 24 h. β-actin was used to assess the quality of total protein loading that statistically differed from the corresponding control values. (C) The level of Hsp90 was decreased by GA and PU-H71. The REST level was dramatically down-regulated in a dose-dependent manner by GA and PU-H71. The levels were compared with the normal control (P<0.05). Densitometry analysis of bands represents the means ± SDs of three independent experiments. Drugs were assayed in triplicate. Errors bars show the SDs of the means. (TIF) [file pone.0220393.s003.tif]

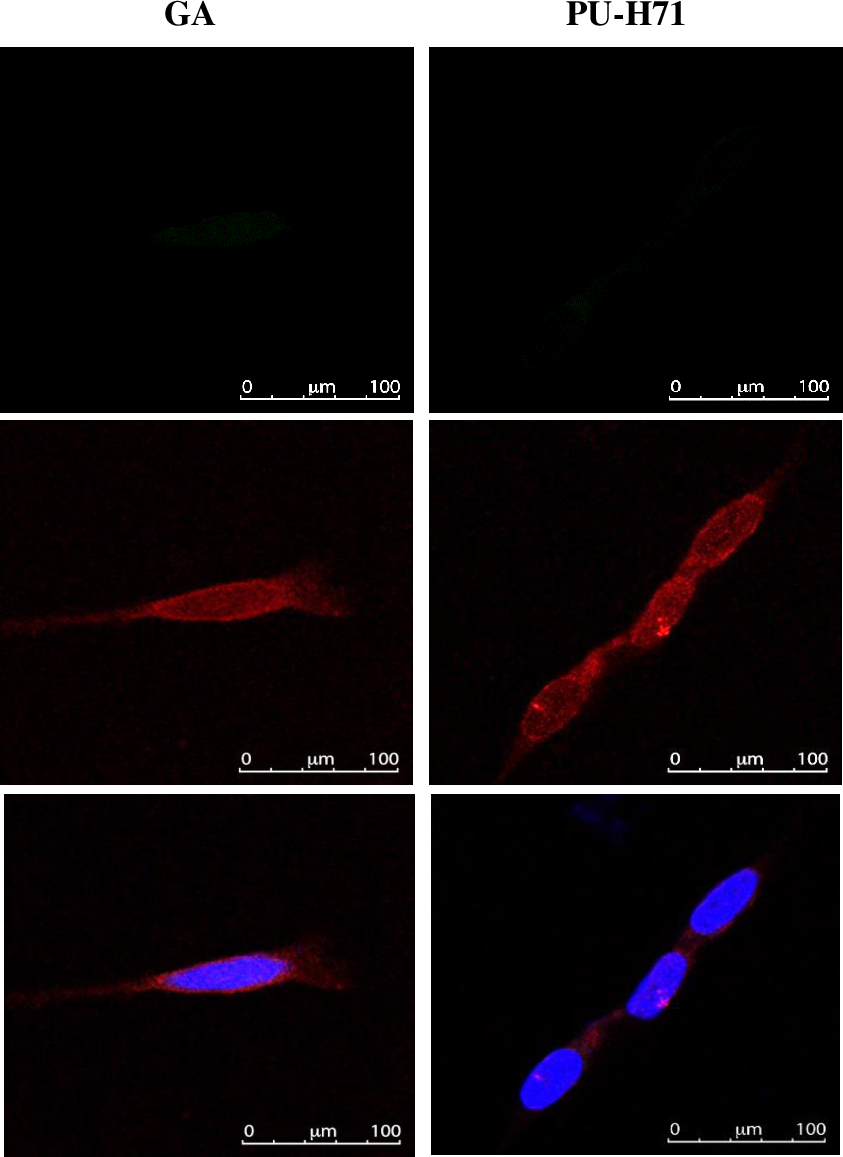

Supplement: S3 Fig — GFP-Htt-480-17Q or GFP-Htt-480-68Q fluorescence (green), REST in red and DNA stained DAPI (blue). Cells transfected with expression vector 17Q or 68Q and treated with GA or PUH-71 presented similar cell protection results; decreased mHtt and REST level, and they had protective effects against mHtt toxicity, decreased mHtt in nucleus and REST level. (TIF) [file pone.0220393.s004.tif]
